# Declines in psychiatric care in inpatient settings in Israel mirror global trend

**DOI:** 10.1186/2045-4015-2-30

**Published:** 2013-08-15

**Authors:** Susan H Busch, Dominic Hodgkin

**Affiliations:** 1 Department of Health Policy and Management, Yale School of Public Health, 60 College Street, Suite 300B, New Haven, CT 06520, USA; 2 The Heller School for Social Policy and Management, Brandeis University, Heller-Brown Building 264, Waltham, MA 02454, USA

## Abstract

Levinson and Lerner provide compelling evidence that reforms to the mental health system in Israel led to significant declines in institutional-based care. These declines are similar to those found in other high income countries over the same time period. Additional evidence on concurrent changes to the amount and quality of care in community settings is an important area for future research.

## 

In their recent IJHPR paper Levinson and Lerner [1] examine the effects of a series of reforms to the mental health system in Israel on inpatient care use. They are particularly interested in whether the structural reform of psychiatric hospitals that occurred in 2001 hastened the movement of psychiatric patients from inpatient care to community based care. They examine a longer time period than prior work (through 2010) and conduct septe analyses of individuals with schizophrenia and individuals with affective disorders.

The authors’ findings are noteworthy. As expected, they find steep declines in mental health care provided in institutional settings. For example, between 2000 and 2011 the number of inpatient days for first-in-life patients with schizophrenia declined by 44 percent and between 2000 and 2009 the rate of first-in-life admissions for schizophrenia declined by 35 percent. The declines in inpatient care for first-in-life patients with affective disorders were much less steep. Similar results were found for length of stay. The authors argue these declines were due to reduced demand resulting from increased availability of care in community settings. They base this conclusion, in part, on the continued decline in inpatient days in the latter half of this study (from 2006 through 2010), even though there was no additional reduction in the number of beds during this time period. The authors argue that if the decline in inpatient use had been only due to a lack of beds, this continued decline would not have occurred. These results suggest the reforms in Israel had the desired effect of reducing reliance on costly inpatient care for individuals with mental illness.

The shift away from the inpatient setting for treatment for mental health disorders is not unique to Israel. The number of psychiatric beds per capita fell sharply in all Western European countries between 1978 and 2002 [2]. Data from the WHO Mental Health Atlas Project 2011 allow us to compare the number of beds dedicated to mental health care in Israel to other high income countries (as defined by World Bank income groups). These data indicate that Israel is close to the median of these countries. Among World Bank high income group countries, the median number of psychiatric beds per capita (including both mental health hospitals and psychiatric beds in general hospitals) was 44.5 per 100,000 [3], while in Israel the number of beds per capita was 45 per 100,000 in 2010 [1]. This figure shows considerable decline from Israel’s beds per capita figure in 2001. Interestingly, before the latest decline (in 2001), Israel’s beds per capita was close to the median for higher income countries (87 per 100,000) [4]. This suggests that Israel’s recent decline in beds ran pllel to trends elsewhere.

The global movement away from long-term treatment for mental health in institutional settings was encouraged for many reasons, including increased concerns about the human rights of individuals with mental health disorders, increased perceived efficiency or cost effectiveness of alternate care settings, and innovation in the form of new effective prescription medications that facilitate living in the community. Yet, most believe a balanced approach is needed with some short term care provided in inpatient settings, along with an array of available community services.

This suggests that a more difficult question is whether Israel currently has achieved *optimal* levels with regard to its bed to population ratio, admission rate, and average length of stay. Among Western European countries, there is substantial variation in the number of psychiatric beds per capita (although this range has narrowed with the decline in beds). One might expect that the optimal number of beds per capita is likely to differ by country (and even region) depending on both contextual and cultural factors. A full evaluation of the effect of the reforms in all treatment settings is beyond the scope of the Levinson and Lerner paper, but their findings suggest there is a need for more work in this area.

Levinson and Lerner do cite two measures to reassure readers that declines in institutional-based care are not so dramatic that they have resulted in an overall decline in quality of care. First, they find that post reform, there was a decline in the percentage of patients who were readmitted to psychiatric inpatient care within three years of discharge. Second, they note that the proportion of admissions that were compulsory has increased only slightly (from 24 to 28 percent) over the time period studied. If quality had declined dramatically, such that individuals were being discharged significantly sicker without adequate community support, one would have expected greater increases in both these measures.

While these measures provide some reassurance, more granular information will be necessary before it can be definitively claimed that the de-institutionalization experienced in Israel was an unqualified success or that no additional declines in inpatient use would be beneficial. For example, although it is beyond the scope of this paper, our understanding of the reform would be enhanced by additional data on the types and intensity of community-based services available, whether these services are geographically located near populations with need, changes in patient quality of life and satisfaction with services, overall changes to the level of global resources devoted to mental health care, and the level of coordination and integration among mental health providers and housing and vocational rehabilitation agencies. Additional studies using these and other measures would provide further valuable information about the context of reform and the effects on individuals with mental health disorders.

## Competing interests

The authors declare that they have no competing interests.

## Authors’ information

Susan H Busch is a health economist and Associate Professor of Health Policy at the Yale School of Public Health. Her research focuses on insurance design for treatment of mental health and substance use disorders.

Dominic Hodgkin is an economist and Associate Professor at Brandeis University. His research focuses on the effects of different organizing and financing approaches in health care, particularly addressing mental health and substance abuse treatment.

## Commentary on

“Hospitalization of patients with schizophrenic and affective disorders in Israel in the aftermath of the structural and rehabilitation reforms”.
